# The Beginnings of Modern Research on Propolis in Poland

**DOI:** 10.1155/2013/983974

**Published:** 2013-06-23

**Authors:** Andrzej K. Kuropatnicki, Ewelina Szliszka, Małgorzata Kłósek, Wojciech Król

**Affiliations:** ^1^Pedagogical University of Krakow, Karmelicka 41, 31-128 Krakow, Poland; ^2^Department of Microbiology and Immunology, Medical University of Silesia in Katowice, Jordana 19, Rokitnica, 41-808 Zabrze, Poland

## Abstract

Propolis studies in Poland were initiated by Professor Stan Scheller in the 1960s. It was a team of Polish researchers who developed a method of introducing hydrophobic ethanol extracts of propolis (EEP) into aqueous solutions, which enabled the study of their biological properties. The studies performed in Poland showed that EEP possesses antioxidant, radioprotective, and immunostimulating properties. It was possible to demonstrate antibacterial activity of propolis on Gram-positive bacteria, virulent *Mycobacterium tuberculosis*, and protozoa as well as stimulating activity of aqueous extracts of propolis on proliferation of cells *in vitro*. Polish investigators showed that propolis stimulates regeneration of tissue, acts as antioxidant and radioprotector, has strong immunostimulative properties, affects animals' life span by extending it, and improves intellectual and life functions of the elderly.

In Poland propolis studies have a long, over a 50-year-old tradition. They were initiated in the early 1960s in the Chair of Microbiology and Immunology of the Medical School in Zabrze, which is one of the two medical schools of the present Medical University of Silesia in Katowice. Propolis studies were a brainchild of the late Professor Jerzy Szaflarski, then Head of Chair of Microbiology in Zabrze, a veterinary doctor by profession. Prof. Szaflarski passed his interest in propolis on to his young assistants, Dr. Stanisław Scheller (see Figure 1), who suspended his own studies on tuberculosis and got involved in propolis studies, and Dr. Leszek Ilewicz, a dentist, who as a visiting researcher had spent some time in the former Soviet Union. Dr. Ilewicz brought the results of Soviet studies on propolis to Poland. 

Stanisław Scheller (1928–2002) was born in Lvov (then Poland) where he lived and completed his school education. In 1947 he took up veterinary studies at Wroclaw University. In 1950, as a student, he began working as a junior assistant of Prof. Ludwik Hirszfeld in the Department of Medical Microbiology at the Medical Academy of Wrocław. In that time he focused his interests on blood groups in dogs [1] and then on leptospirae and leptospirosis in man and animals. The results of his studies were presented in his doctoral dissertation prepared under the supervision of Prof. Józef Zwierz in 1961 [2]. Ten years later, Scheller joined the Department of Microbiology at the Silesian Medical Academy in Zabrze as a senior assistant. His many years of research on tuberculosis resulted in a postdoctoral dissertation on tuberculosis resistance using *in vitro* cell culture [3–6]. In 1966, inspired by Prof. Szaflarski (Head of the department), he dedicated himself to studies of propolis, the effects of which he experimented many times on himself. In 1997 Stanisław Scheller changed his first name to Stan.

Prof. Scheller's interest lay in unconventional methods of treatment. He strongly believed in alternative medicine and its potential in supporting conventional methods of treatment. In his opinion, natural products, natural healing processes, and complementary therapies assist a physician in his conventional management of disease. Scheller endeavored to apply natural products in cases where conventional medicine was shown to be ineffective. 

In 1967, under his supervision, a series of experiments were performed in which propolis as a raw material was extracted with ethanol and after evaporation of alcohol the extract obtained was dispersed in Tween 20 saline solution. This method of introducing hydrophobic ethanol extracts of propolis (EEP) into aqueous solutions enabled the study of their biological properties and medical use. It must be emphasized that it was Scheller who popularized EEP. He proved bactericidal activity of EEP against Gram-positive, virulent *Mycobacterium tuberculosis,* and some protozoa. A group of Polish scientists under his guidance confirmed the activation of metabolic processes of cells cultured *in vitro* and regeneration of experimentally damaged tissue (bone, cartilage, and tooth pulp) or diseased tissues. They showed antioxidant activity of EEP as well as its immune-stimulating and radioprotective properties [7]. 

The interests of Polish scientists of Silesian Medical Academy in propolis resulted in a series of articles published in Poland and abroad. In 1964 the data on the studies and laboratory evaluation of alcohol extract of propolis together with some observations concerning its therapeutic properties were published [8–10]. Four years later antibacterial properties of propolis were presented [11] and then in 1971 preliminary evaluation of clinical usefulness of propolis preparations [12]. Further investigations provided topic for the next series of scientific articles, which were published in eleven parts in *Arzneimittelforschung/Drug Research* under the common title “Biological properties and clinical application of propolis” [13–23]. 

In early studies 19 fractions of EEP were isolated and main attention was directed to their antibacterial properties [13]. Further research dealt with the antiprotozoal activity of propolis. It was shown that ethanolic extract of propolis acts effectively against *Trichomonas vaginalis *and *Toxoplasma gondii* [14]. Other studies which focused on *Staphylococcus *strain bacteria showed that EEP breaks the resistance of bacteria [15]. In studies on cells cultured *in vitro* it was observed that EEP when added to culture medium with grown cells caused intense activation of mitoses. What is more, EEP triggered activation of NADH2 reductase in cell culture [16]. Experimenting on laboratory animals Scheller's team showed activation of glucose-6-phosphatase, NADPH2, and tetrazolium reductase in rats after application of EEP [17]. 

Positive results of EEP activity upon cells *in vitro* were insufficient to prove its regenerative activity. The proof lay in experiments conducted on animals. In order to obtain possibly high credibility, tissues hard to regenerate were chosen, namely, bone, articular cartilage, and dental pulp. Application of EEP accelerated the healing processes in damaged cartilage as well as enhanced ossification in an artificially induced bone defects. Furthermore, it was proved that EEP inserted into the joint was well tolerated [18, 19]. It was shown that EEP supported regeneration of dental pulp and reduced inflammatory and degenerative processes [20, 24, 25]. 

The studies conducted for several years (unfortunately, the results have never been published) showed that biological properties of EEP greatly depend on several ecological factors, including geographical region, plant source, season, and a method of harvesting. In that time Scheller established a permanent cooperation with a reputable beekeeper Jan Batko whose apiary was located in a green corner of South Poland (the Carpathian Mountains) near the village of Kamianna. The samples of propolis obtained from Batko were used to study antibacterial activity of EEP against reference strain *Staphylococcus aureus* Oxford P209. It was found out that antibacterial activities of EEP samples collected in various years were different which led to conclusion that the samples differed in their chemical composition. Dr. Wiesława Maciejewicz from Medical Academy in Lublin, Poland, took up studies on chemical composition of EEP. In a sample of defined bacteriostatic activity a number of sesquiterpenes were found [26]. Unfortunately, the cooperation was soon ceased and did not result in chemical standardization of Polish propolis.

Basic studies which confirmed EEP activity were conducted simultaneously with clinical studies on humans at university hospitals. EEP having various vinculums was used in 150 cases of postsurgical complications and burns. It was noted that, when using EEP, significantly better results were obtained in comparison to conventional methods. The fact was reflected in better physiological healing and shortening of the treatment time by about 50–80% [27]. The observed anti-inflammatory effect in the treatment of wounds was confirmed in studies on patients with chronic bronchitis [28, 29]. Use of EEP as well as crude propolis improved biochemical and immunological indicators in geriatric patients [30, 31]. 

In cooperation with orthopedic surgeons a study was conducted on osteonecrosis of thigh bone. The study involved 54 cases of hip joint with aseptic necrosis of thigh bone. EEP was administered intra-articularly into 22 hips, and no typical conservative treatment was conducted. In the remaining 32 cases, various forms of treatment were used. The obtained results confirmed the purpose of enriching conservative treatment with intra-articular injections of EEP, especially in advanced stages of necrosis (III-IV period of illness) and also in those patients whose parents did not give their consent to surgery in an early stage of the illness [32]. 

In Zabrze, preliminary pharmacological investigations of propolis were initiated. The effects of EEP on respiration and blood pressure in rats were studied. The animals, male BALB/c mice and male rats of the Wistar strain, were premedicated with amphetamine and then EEP was injected intraperitoneally. The extract of propolis was shown to have a low impact on the overall condition of the experimental animals [22]. Ethanolic extract of propolis was also shown to be capable of increasing the number of plaque-forming cells in spleens of immunized male BALB/c mice, demonstrating their ability to produce antibodies. It was found out that the single EEP dose exerting the maximal plaque formation (a three-fold increase over control) was 500 *μ*g/mouse. When this dose was repeated within 24 hours, the plaque-producing effect was even stronger; however, further increases of the propolis dose or the number of its administrations had an inhibitory effect on the plaque formation. The authors concluded that the time interval between EEP administration and the immunization process should not exceed 48 hours [33].

In other experiments EEP was administered parenterally to rabbits. It was shown that EEP did not induce the synthesis of antibodies, what was confirmed by immunoassay tests such as complement fixation test or double diffusion gel precipitation [19]. 

Scheller's team cooperated with other university departments and research institutes. In collaboration with nuclear physicists they performed neutron analysis which allowed for the determination of 30 trace elements in propolis [34], whereas chromatographic analysis made it possible to determine several free amino acids, beside arginine and proline. Scheller knew that propolis stimulates mammalian tissue regeneration and suggested that proline and arginine must play a key role in this process. He also suggested that arginine enhances protein biosynthesis and ability to stimulate mitosis. Proline promotes the formation of elastin and collagen, which are constituents of the connective tissue. The results were published in 1986 [35]. 

In 1982 a laboratory for photon emission measurements was founded in the department. Granulocytes engaged in the phagocytosis of opsonized zymosan emit light by a process that is inhibited by superoxide dismutase and catalase. Luminous animals have been known since ancient times; however, “artificial” chemiluminescence (CL) was first described in 1877 by Radziszewski, a Professor of Chemistry at Lemberg in Galicia. Radziszewski observed the yellow light emission when oxygen was bubbled into an alkaline ethanolic solution of 2,4,5-triphenylimidazole (lophine). Fifty years later, Albrecht reported the luminescent properties of 5-amino-2,3,-dihydrophthalazine-1,4-dione (luminol). Luminol is a very lipid-soluble substance that can penetrate cells and tissue easily, and it is used in various ways to measure luminescence in single phagocytic cells, groups of cells, and cells bound to or located with tissue. The use of luminol-dependent CL may prove valuable as a method to measure the earliest events in the inflammatory process and may facilitate studying the mechanisms that produce inflammation. Luminol-dependent CL predominantly reflects the production of H_2_O_2_ together with nitric oxide (NO) peroxynitrite formation [36].

In 1986 during the 5th meeting of Polish Immunological Society the first results of inhibition by EEP of luminol-dependent CL human neutrophils stimulated by opsonized zymosan were presented [37]. These studies initiated a series of *in vitro* studies on antioxidant and anti-inflammatory properties of propolis and its components that were carried out by Scheller's team [38–42]. The paper “Effects of ethanol extract of propolis (EEP) and its flavones on inducible gene expression in J774A.1 macrophages” published in the Journal of Ethnopharmacology in 2004 was the very last publication of Prof. Scheller [42].

In another study, Scheller's team tested 19 phenolic compounds present in propolis for their anti-inflammatory activity. The luminol-enhanced chemiluminescence generated by neutrophils that had been stimulated by phorbol myristate acetate was evaluated. It was found that caffeic-acid-phenylethyl-ester abolished the chemiluminescence completely at a concentration of 10 *μ*M, while three flavone derivatives and three flavonols (galangin, kaempferol, and kaempferide) diminished this chemiluminescence by 73–93% at the same concentration. These results indicate that some of the phenolic components of the ethanol extract of propolis are active in exerting its renowned anti-inflammatory activity [41].

Other studies conducted in Zabrze were to provide an answer to the question whether propolis extracts or its isolated compounds can act directly upon tumour cells. To do this, cytotoxic cells were stimulated *in vivo,* and then their ability to destroy target cells, in this case tumour cells, was examined. In several trials an increase of lytic abilities of so stimulated cells was obtained. Within studies of EEP effect on tumour cells, mice infected intraperitoneally with Ehrlich cancer were subjected to experiments in three groups. Mice in the first group were injected with EEP, in the second group with bleomycin, and in the third with bleomycin together with EEP. The survival rate of the mice was 55% after EEP, 40% after bleomycin, and the mice in the third group demonstrated the shortest survival. In conclusion the authors say that the antitumor effect of propolis was due to the flavonoids inhibiting the incorporation of thymidine, uridine, and leucine into the carcinoma cells, thus leading to an inhibition of DNA synthesis [43]. 

Interesting studies were conducted in 1989 prior to Chernobyl disaster. In these studies EEP was tested as a protective agent against gamma irradiation in mice. The mice were exposed to sublethal doses of gamma irradiation from a ^60^Co source and treated intraperitoneally with EEP which was administered before and after irradiation. It was observed that approximately 90% of experimental animals survived without showing any changes in their appearance or behavior nor any signs of immunosuppression. Their leukocyte count as well as their spleens' plaque-forming activity returned to normal. It was suggested that an antioxidant and a free radical scavenger in the EEP are responsible for the radiation protective effect of the extract of this natural product [44]. A series of experiments performed in Zabrze have proven that EEP administered orally to animals extends their lives statistically significantly [45]. 

Other research confirmed the antioxidant properties of EEP. The free radical scavenging ability of EEP was demonstrated by electron spin resonance spectroscopy, when DPPH (2,2-diphenyl-1-picrylhydrazyl) was treated with increasing concentrations of EEP [46]. Also antibacterial properties of EEP were confirmed. Ethanolic extract of propolis was incubated with 8 different antibiotics: penicillin G, doxycycline, streptomycin, cloxacillin, chloramphenicol, cefradine, ampicillin, and polymyxin B. Culture medium contained additionally a strain of *Staphylococcus aureus*. It was demonstrated that EEP had a synergistic effect on the antibacterial activity of cloxacillin and streptomycin [47].

A correlation between virulence of various strains of mycobacteria and their susceptibility to ethanolic extract of propolis was the subject of study conducted on 17 different strains of mycobacteria. The studies showed that out of the 13 virulent strains seven were susceptible and six were resistant to EEP. The remaining 4 nonvirulent strains were not susceptible to EEP. The results showed that although there was no full correlation between virulence of the mycobacteria and their susceptibility to EEP, a few strains that were resistant to EEP were nonvirulent [48, 49].

Scheller's team cooperated with a number of research centers in Poland and in the world. In 1995 Polish scientists from Zabrze established cooperation with Dr. Tetsuya Matsuno of the National Institute of Infectious Diseases and Rindai Yamamoto (Nihon Natural Therapy Co., Ltd) in Japan. The following year Scheller visited Japan where he presented the results of his research. This gave rise to further meetings of Polish researchers who participated in Japan-Poland international seminars on propolis in 2006, 2008 and 2010.

At present Prof. Król's team focuses on cytotoxic effect of propolis in combination with tumor necrosis factor-related apoptosis-inducing ligand (TRAIL), which is a naturally occurring anticancer agent that preferentially induces apoptosis in cancer cells. The cytotoxic and apoptotic effect of EEP and phenolic compounds identified in propolis were studied in combination with TRAIL using HeLa cancer cells. The study demonstrated that EEP and its components significantly sensitize to TRAIL-induced death in cancer cells. The percentage of the apoptotic cells after exposure to 50 *μ*g/mL EEP and 100 ng/mL TRAIL increased to 71.10 ± 1.16%. Apigenin and CAPE at the concentrations of 50 *μ*M (58.87 ± 0.75% and 49.59 ± 0.39%, resp.) exhibited the strongest cytotoxic effect in combination with TRAIL on HeLa cells. It was shown that EEP markedly augmented TRAIL mediated apoptosis in cancer cells, and the importance of propolis in chemoprevention of malignant tumors was confirmed [50]. In another work, it was demonstrated that EEP markedly augmented TRAIL-mediated apoptosis in prostate cancer cells [51]. The results of these studies together with data obtained by other researchers have been summarized in a review [52]. 

Summing up, the studies conducted in the Chair of Microbiology and Immunology in Zabrze made it possible to determine for the first time and in adequate way

1. antibacterial activity of propolis on Gram-positive bacteria, virulent *Mycobacterium tuberculosis,* and protozoa,
2. stimulating activity of aqueous extracts of propolis on proliferation of cells *in vitro*, without signs of pathologic stimulation, such as chromosomal aberration.

It was shown without any doubt that propolis

1. stimulates regeneration of experimentally damaged tissue as well as tissue in pathological processes,
2. acts as antioxidant,
3. acts radioprotectively,
4. has strong immunostimulative properties,
5. has extracts that have cytolytic activity on anticancer cells in animal studies,
6. affects animals' life span by extending it,
7. improves intellectual and life functions of the elderly,
8. speeds up recovery in patients with prostatitis [53].

## Figures and Tables

**Figure 1:**
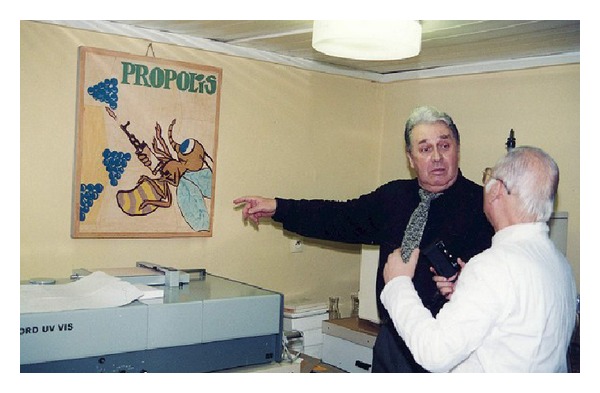
Professor Scheller in laboratory.

## References

[1] Scheller S, Mycielski R (1953). Blood groups in dogs. *Archiwum Immunologii i Terapii Doświadczalnej*.

[2] Scheller S (1962). Wpływ aminokwasów na wzrost L.icterohaemorrhagiae. *Medycyna Weterynaryjna*.

[3] Scheller S, Szurman J, Szaflarski J (1964). Phagocytoze und Wachstum der Tuberkelbakterien in HeLa Zellkulturen. *Zentralblat für Bakteriologie, Parazitenkunde, Infektionskrankheiten und Hygiene*.

[4] Scheller S, Szurman J, Urbańska L (1964). Untersuchungen über Phagozytose und Wachstumeigenschaften der photochromogenen Mykobakterien in HeLa Zellkulturen. *Zentralblat für Bakteriologie, Parazitenkunde, Infektionskrankheiten und Hygiene*.

[5] Urbańska L, Scheller S (1967). Cell culture in vitro as an experimental model for investigation of certainbiological properties of acid-fastm mycobacteria. *Polish Medical Journal*.

[6] Scheller S (1969). Badania na odpornością w gruźlicy metodą hodowli komórek in vitro. *Biuletyn Służby Sanitarno-Epidemiologicznej Województwa Katowickiego*.

[7] Król W (2002). Stan Scheller (1928–2002): lekarz mikrobiolog. Profesor doktor habilitowany nauk medycznych. *Biuletyn Informacyjny ŚAM*.

[8] Furowicz A, Ilewicz L, Stojko A, Szaflarska-Stojko E, Zaleski W (1964). Badania doświadczalne i obserwacje kliniczne przy zastosowaniu alkoholowego roztworu propolisu. *Przegląd. Stomatologiczny*.

[9] Furowicz A, Ilewicz L, Zachaczewska M, Zaleski W (1964). Kit pszczeli i jego właściwości lecznicze. *Życie Weterynaryjne*.

[10] Furowicz A, Ilewicz L, Stojko A, Szaflarska-Stojko E (1965). Kit pszczeli i jego właściwości lecznicze. *Życie Weterynaryjne*.

[11] Scheller S, Rogala D, Stasiak E, Żurek H (1968). Antibacterial properties of propolis. *Polskie Archiwum Weterynarii*.

[12] Chorążak T, Szaflarski J, Seferowicz E, Scheller S (1971). Preliminary evaluation of clinical usefulness of propolis (beeswax) preparations. *Przegląd Lekarski*.

[13] Scheller S, Szaflarski J, Tustanowski J, Nolewajka E, Stojko A (1977). Biological properties and clinical application of propolis. I. Some physico chemical properties of propolis. *Arzneimittel-Forschung*.

[14] Starzyk J, Scheller S, Szaflarski J, Moskwa M, Stojko A (1977). Biological properties and clinical application of propolis. II. Studies on the antiprotozoan activity of ethanol extract of propolis. *Arzneimittel-Forschung*.

[15] Scheller S, Tustanowski J, Kuryło B (1977). Biological properties and clinical application of propolis. III. Investigation of the sensitivity of staphylococci isolated from pathological cases to ethanol extract of propolis (EEP). Attempts on inducing resistance in laboratory staphylococcus strain to EEP. *Arzneimittel-Forschung*.

[16] Scheller S, Nolewajka E, Panasiewicz M, Dziekanowska D, Tustanowski J, Szaflarski J (1977). Biological properties and clinical application of propolis. IV. The action of ethanol extract of propolis (EEP) on cells cultured in vitro. *Arzneimittel-Forschung*.

[17] Kamiński M, Scheller S, Nolewajka E (1977). Biological properties and clinical application of propolis. V. The action of ethanol extract of propolis (EEP) on laboratory animals. Histochemical investigations. *Arzneimittel-Forschung*.

[18] Scheller S, Stojko A, Szwarnowiecka I, Tustanowski J, Obuszko Z (1977). Biological properties and clinical application of propolis. VI. Investigation of the influence of ethanol extracts of propolis (EEP) on cartilaginous tissue regeneration. *Arzneimittel-Forschung*.

[19] Scheller S, Tustanowski J, Feluś E, Stojko A (1977). Biological properties and clinical application of propolis. VII. Investigation of immunogenic properties of ethanol extract of propolis (EEP). *Arzneimittel-Forschung*.

[20] Stojko A, Scheller S, Szwarnowiecka I, Tustanowski J, Ostach H, Obuszko Z (1978). Biological properties and clinical application of propolis. VIII. Experimental observation on the influence of ethanol extract of propolis (EEP) on the regeneration of bone tissue. *Arzneimittel-Forschung*.

[21] Scheller S, Ilewicz L, Luciak M, Skrobidurska D, Stojko A, Matuga W (1978). Biological properties and clinical application of propolis. IX. Experimental observation on the influence of ethanol extract of propolis (EEP) on dental pulp regeneration. *Arzneimittel-Forschung*.

[22] Kleinrok Z, Borzęcki Z, Scheller S, Matuga W (1978). Biological properties and clinical application of propolis. X. Preliminary pharmacological evaluation of ethanol extract of propolis (EEP). *Arzneimittel-Forschung*.

[23] Scheller S, Luciak M, Tustanowski J, Kozioł M, Obuszko Z, Kuryło B (1978). Biological properties and clinical application of propolis. XI. Histopathological analysis after intravenous application of ethanol extract of propolis (EEP). *Arzneimittel-Forschung*.

[24] Ilewicz L, Luciak M, Skrobidurska D, Scheller S, Stojko A (1979). Działanie etanolowych ekstraktów propolisu na miazgę zębową u psów. *Czasopismo Stomatologiczne*.

[25] Scheller S, Owczarek S, Król W, Malinowska B, Nikodemowicz E, Aleksandrowicz J (1989). Immunisierungsversuche bei zwei Fallen von Alveolitis fibroticans bei Abnehmer Leistungsfähigkeit des Immunsystems unter Anwendung von Propolis-Ethanolextract (EEP). *Heilkunst*.

[26] Maciejewicz W, Scheller S, Daniewski M (1983). Gas chromatography—mass spectrometry investigation of propolis. Analysis of sesquiterpenes. *Acta Poloniae Pharmaceutica*.

[27] Scheller S, Zapotoczny S, Kubacka S, Tustanowski J, Szyszko S (1980). Zastosowanie etanolowego ekstraktu propolisu w chirurgii. *Przegląd Lekarski*.

[28] Pawlak F, Scheller S, Kieloch-Szkoda M, Kozioł M (1983). Zastosowanie etanolowego ekstraktu propolisu (EEP) u chorych na przewlekłe zapalenie oskrzeli. *Balneologia Polska*.

[29] Scheller S, Król W, Żydowicz G (1995). Ethanol extract of propolis (EEP) and Dolomite potentiates the immunstimulatory effect of Biostymine and Levamisole in chronic bronchitis. *Pharmacology Life Sciences*.

[30] Scheller S, Pawlak F, Kokoszka J (1984). Die Anwendung von Propolisethanolextraktes (EEP) in der Geriatrie. *Die Heilkunst*.

[31] Frankiewicz L, Scheller S (1984). Bienen-Kittharz stimuliert das Immunsystem. *Ärztliche Praxis*.

[32] Przybylski J, Scheller S (1985). Frühzeitige Ergebnisse der Behandlung von Legg-Calve-Perthes Krankheit mittels Gelenkinjektionen von wässriger Extrakten von Propolis. *Zeitschrift für Orthopädie und ihre Grenzgebiete*.

[33] Scheller S, Gazda G, Pietsz G (1988). The ability of ethanol extract of propolis to stimulate plaque formation in immunized mouse spleen cells. *Pharmacological Research Communications*.

[34] Scheller S, Czauderna M, Król W (1989). Trace elements in propolis and its ethanolic extract (EEP) as determined by neutron activation analysis. *Zeitschrift für Naturforschung C*.

[35] Gabryś J, Konecki J, Król W, Scheller S, Shani J (1986). Free amino acids in bee hive product (propolis) as identified and quantified by gas-liquid chromatography. *Pharmacological Research Communications*.

[36] Dodeigne C, Thunus L, Lejeune R (2000). Chemiluminescence as a diagnostic tool. A review. *Talanta*.

[37] Król W, Czuba ZP, Scheller S (1986). Effect of ethanol extract of propolis and flavonols as chemiluminescence of human neutrophils. *Immunologia Polska*.

[38] Król W, Czuba Z, Scheller S, Gabryś J, Grabiec S, Shani J (1990). Anti-oxidant property of ethanolic extract of propolis (EEP) as evaluated by inhibiting the chemiluminescence oxidation of luminol. *Biochemistry International*.

[39] Czuba Z, Król W, Scheller S, Shani J (1992). Effect of cinnamic and acrylic acids’ derivatives on luminol-enhanced chemiluminescence of neutrophils. *Zeitschrift fur Naturforschung C*.

[40] Król W, Shani J, Czuba Z, Scheller S (1992). Modulating luminol-dependent chemiluminescence of neutrophils by flavones. *Zeitschrift fur Naturforschung C*.

[41] Król W, Scheller S, Czuba Z (1996). Inhibition of neutrophils’ chemiluminescence by ethanol extract of propolis (EEP) and its phenolic components. *Journal of Ethnopharmacology*.

[42] Błońska M, Bronikowska J, Pietsz G, Czuba ZP, Scheller S, Król W (2004). Effects of ethanol extract of propolis (EEP) and its flavones on inducible gene expression in J774A.1 macrophages. *Journal of Ethnopharmacology*.

[43] Scheller S, Król W, Świącik J, Owczarek S, Gabryś J, Shani J (1989). Antitumoral property of ethanolic extract of propolis in mice-bearing Ehrlich carcinoma, as compared to bleomycin. *Zeitschrift fur Naturforschung C*.

[44] Scheller S, Gazda G, Król W (1989). The ability of ethanolic extract of propolis (EEP) to protect mice against gamma irradiation. *Zeitschrift fur Naturforschung C*.

[45] Scheller S, Król W, Sedlaczek R, Żydowicz G, Wójcik L, Shani J (1994). Ethanolic extract of propolis (EEP), a natural antioxidant, prolongs life span of male and female mice. *Pharmacology Life Sciences*.

[46] Scheller S, Wilczok T, Imielski S, Król W, Gabryś J, Shani J (1990). Free radical scavenging by ethanol extract of propolis. *International Journal of Radiation Biology*.

[47] Król W, Scheller S, Shani J, Pietsz G, Czuba Z (1993). Synergistic effect of ethanolic extract of propolis and antibiotics on the growth of *Staphylococcus aureus*. *Arzneimittel-Forschung*.

[48] Scheller S, Kawalski H, Oklek K (1998). Correlation between virulence of various strains of Mycobacteria and their susceptibility to ethanolic extract of propolis (EEP). *Zeitschrift fur Naturforschung C*.

[49] Scheller S, Dworniczak S, Waldemar-Klimmek K, Rajca M, Tomczyk A, Shani J (1999). Synergism between ethanolic extract of propolis (EEP) and anti-tuberculosis drugs on growth of *Mycobacteria*. *Zeitschrift fur Naturforschung C*.

[50] Szliszka E, Czuba ZP, Domino M, Mazur B, Żydowicz G, Król W (2009). Ethanolic extract of propolis (EEP) enhances the apoptosis-inducing potential of TRAIL in cancer cells. *Molecules*.

[51] Król W, Szliszka E, Czuba ZP, Bronikowska J, Mertas A, Paradysz A (2011). Ethanolic extract of propolis augments TRAIL-induced apoptotic death in prostate cancer cells. *Evidence-Based Complementary and Alternative Medicine*.

[52] Szliszka E, Król W (2013). Polyphenols isolated from propolis augment TRAIL-induced apoptosis in cancer cells. *Evidence-Based Complementary and Alternative Medicine*.

[53] Scheller S, Rawski W, Gazda G, Pietsz G (1985). Behandlungsversuche der chronischen Prostatentzündung mittels simultaner Anwendung von Polyvakzin, Decaris (Levamisole) und Äthanol-Extract von Propolis (EEP). *Die Heilkunst*.

